# Spatial and temporal variation in proximity networks of commercial dairy cattle in Great Britain

**DOI:** 10.1016/j.prevetmed.2021.105443

**Published:** 2021-09

**Authors:** Helen R. Fielding, Matthew J. Silk, Trevelyan J. McKinley, Richard J. Delahay, Jared K. Wilson-Aggarwal, Laetitia Gauvin, Laura Ozella, Ciro Cattuto, Robbie A. McDonald

**Affiliations:** a Environment and Sustainability Institute, University of Exeter, Penryn Campus, Penryn, TR10 9FE, UK; b College of Medicine and Health, University of Exeter, Exeter, EX2 5DW, UK; c National Wildlife Management Centre, Animal and Plant Health Agency, Sand Hutton, York, YO41 1LZ, UK; d ISI Foundation, Via Chisola 5, 10126, Torino, Italy; e Computer Science Department, University of Turin, Corso Svizzera 185, 10149, Torino, Italy

**Keywords:** Cattle, Social network, Contact, Disease transmission, Livestock

## Abstract

The nature of contacts between hosts can be important in facilitating or impeding the spread of pathogens within a population. Networks constructed from contacts between hosts allow examination of how individual variation might influence the spread of infections. Studying the contact networks of livestock species managed under different conditions can additionally provide insight into their influence on these contact structures.

We collected high-resolution proximity and GPS location data from nine groups of domestic cattle (mean group size = 85) in seven dairy herds employing a range of grazing and housing regimes. Networks were constructed from cattle contacts (defined by proximity) aggregated by different temporal windows (2 h, 24 h, and approximately 1 week) and by location within the farm.

Networks of contacts aggregated over the whole study were highly saturated but dividing contacts by space and time revealed substantial variation in cattle interactions. Cows showed statistically significant variation in the frequency of their contacts and in the number of cows with which they were in contact. When cows were in buildings, compared to being on pasture, contact durations were longer and cows contacted more other cows. A small number of cows showed evidence of consistent relationships but the majority of cattle did not. In one group where management allowed free access to all farm areas, cows showed asynchronous space use and, while at pasture, contacted fewer other cows and showed substantially greater between-individual variation in contacts than other groups.

We highlight the degree to which variations in management (e.g. grazing access, milking routine) substantially alter cattle contact patterns, with potentially major implications for infection transmission and social interactions. In particular, where individual cows have free choice of their environment, the resulting contact networks may have a less-risky structure that could reduce the likelihood of direct transmission of infections.

## Highlights

• We analysed high resolution proximity networks and GPS locations of dairy cattle.
• Contact rates varied markedly in time and space, and in relation to husbandry.
• Contacts were more frequent and longer in buildings.
• Few cows exhibited evidence of consistent relationships.
• When given free access across the farm, cows showed greater variation in contacts.

## 1 Introduction

Host contact rate is a crucial factor in disease spread within and between groups (VanderWaal and Ezenwa, 2016). Modelling contact rate as a homogeneous process can provide an effective basis to simulate transmission in many circumstances (Anderson and May, 1992). Where contact rates vary markedly between individuals, however, the dynamics of directly transmitted infections might better be predicted using contact or proximity networks (Bansal et al., 2007; Craft, 2015; VanderWaal and Ezenwa, 2016). Populations exhibiting strongly modular contact networks, characterised by divisions among subgroups, can experience altered disease dynamics, including reduced overall infection prevalence (Sumner et al., 2018), ‘structural trapping’ of infections, or increased transmission within subgroups (Sah et al., 2017).

Including empirically-determined contact networks rather than homogeneous mixing in transmission models can alter epidemic predictions. In groups of beef cattle, horses, and dogs, using empirical contact data produced lower estimates of epidemic size and duration, compared to simulating random mixing of individuals (Duncan et al., 2012; Milwid et al., 2019a; Wilson-Aggarwal et al., 2019). Incorporating heterogeneous contact structure into within-farm disease transmission models has enabled the dynamics of other cattle infections, including *Mycobacterium avium* subsp. *paratuberculosis*, *Escherichia coli,* and bovine viral diarrhoea, to be more accurately described (Courcoul and Ezanno, 2010; Marcé et al., 2011; Turner et al., 2008).

High-resolution contact data can be particularly useful in predicting disease dynamics more accurately (Bansal et al., 2010; Silk et al., 2017; Springer et al., 2017). For example, models incorporating temporal dynamics using hourly contact networks recorded in small groups of calves have shown that including this fine-scale temporal variation in contact networks can alter outbreak model predictions, especially for infections with smaller basic reproductive ratios (R_0_), of between 1 and 2 (Chen et al., 2014). Such comprehensive contact data are scarce for commercial dairy farms (Álvarez et al., 2014), which are of particular interest due to their more intensive management systems and larger herd size, which increase the potential for disease incursion and within-herd transmission (Brooks Pollock and Keeling, 2009; Conlan et al., 2012). Small-scale studies give crucial insights into cattle behaviour and welfare issues (Chen et al., 2015; Foris et al., 2018), yet do not fully describe more realistic interactions in a typical herd environment. Two notable studies have collected continuous contact data on commercial dairy farms. The first reported within-housing contacts of six small dairy herds in Switzerland (Gygax et al., 2010), and a second studied one UK commercial dairy farm that used a robotic milking machine during four periods (Boyland et al., 2016). Both studies reported that cattle formed a single, unstructured group, and found evidence of individual social preferences. However, it is unclear if these findings can be extrapolated to farms with different herd-management systems and how contacts might differ when animals are in different types of housing, have choice over their environment, or experience different stocking densities. Due to the rise in popularity of ‘zero-grazing’ herds (Haskell et al., 2006), the merits of indoor vs. outdoor-based systems for keeping cattle have been extensively discussed from a health and welfare perspective. Whilst indoor units may be better able to meet nutritional needs and avoid parasitism, climatic stresses and contact with neighbouring herds, they may be associated with a reduced ability to demonstrate natural oestrus and resting behaviours, and an increase in lameness and mastitis cases (Arnott et al., 2016; Crump et al., 2019; Mee and Boyle, 2020).

To form a broader understanding of cattle interactions over multiple herd-management systems, we recorded proximity among cows living in groups on commercial dairy farms in the south-west of England. While region and season were consistent between farms, our groups represented a range of management practices in terms of their milking regimes, housing, grazing access, and group sizes, all of which might be expected to affect interactions among individuals in the group. To capture potential differences in contacts between locations within a farm, we used a combination of proximity sensor technology and GPS devices to describe the cows’ locations and their interactions. We describe the contact networks of cattle groups at different spatial and temporal scales, comparing networks in buildings with those at pasture, and analysing contact frequencies at differing time aggregations. We predicted that interactions would differ among locations on the farm, specifically that cattle would be more able to express social preferences at pasture, and would have more numerous contacts while in buildings. We focussed on network characteristics that might influence the transmission of pathogens: heterogeneity in contact rates and durations, the formation of sub-groups or ‘communities’, and the relative strength of connections within and between these communities.

## 2 Methods

### 2.1 Farms and cattle management

We deployed collars on nine groups of adult cows within seven commercial dairy herds in the UK. For anonymity, we refer to each group by a descriptive feature of the group (Table 1). For six of the nine groups, we recorded contacts among cows that were kept in a milking group separated from other animals in the overall farm herd, as is common practice in UK herds. Cows in the largest herd were routinely managed in several groups and we recorded contacts in two groups: among the dry cows (Dry) and, separately, among the low-yielding milking cows (Housed). No changes were made to the normal routine of the farms, and so during most deployments, cows were added to, and removed from, groups by the farmers as the cows started or finished their lactations. One exception was the Stable group, which included all adult cows, and where no cows joined or left the group during the study period.

**Table 1:** Details of farm management and data collected from nine groups of cattle on seven dairy farms in Cornwall in Summer and Autumn 2018. Details include breed of cattle, grazing management, milking routine, group sizes, number of collars with GPS and proximity sensors that were deployed, and data quality.

Deployment name	Start month of study	Breed of cattle	Source of replacement cows	Group	Calving system	Cow access to farm locations		Grazing type	No. milkings per day	No. cows in group		Study Period (days)	No. cows with complete proximity data	Percentage of the group with data (of collars deployed)
						Buildings	Pasture			Start	End			
Night-housed	Oct-18	Holstein Friesian	Multiple source farms	Milking	All year round	Milking and night	Day	Rotational grazing	2	98	97	6.77	87	88.8
Strip-grazed	Aug-18	Ayrshire	Home-bred	Milking	All year round	Milking	Day and night	Strip grazing	2	52	54	6.83	44	84.6
Free	Aug-18	Holstein Friesian	Home-bred	Milking	All year round	Access at all times	Access at all times	Free roam of pastures	Automated milking system	60	60	6.85	50	83.3
Rotation 1a	Aug-18	Holstein Friesian / Jersey cross	Home-bred	Milking	Autumn calving	Milking	Day and night	Rotational grazing	1	80	96	6.77	64	80.0
Rotation 1b	Oct-18			Milking		Milking and access at all times	Day and night	Rotational grazing	2	98	112	6.79	87	88.8
Dry	Sep-18	Holstein Friesian	Multiple source farms	Dry	All year round	No access	Day and night	Set stocking	0	33	32	6.76	22	59.5
Housed	Sep-18			Milking		Day and night	No access	NA	2	111	106	6.76	100	90.1
Rotation 2	Oct-18	Holstein Friesian	Home-bred	Milking	All year round	Milking	Day and night	Rotational grazing	2	175	181	7.04	95	53.7
Stable	Sep-18	Mixed	Multiple source farms	All cows	Spring calving	Milking	Day and night	Rotational grazing	1	59	59	6.84	37	64.9
									Mean:	85	89	6.82	65	77.1

Grazing management varied among study groups and ranged between allocation of additional strips of grazing after each milking (Strip-grazed), through rotationally grazing a different field or part of a field after each milking (Rotation 1a, Rotation 1b, Rotation 2, Stable, Night-housed), to set stocking in one field (Dry) and free range of all fields on the farm (Free). Variation between farms in this study reflected the diversity of typical practices employed in UK dairy herds at the time of the study.

In general, milking groups were kept on a specific area of pasture and brought into buildings only for milking, with four exceptions: 1) the Housed group were kept in cubicle housing throughout the study, 2) the Night-housed group were in cubicle housing at night, 3) the Rotation 1b group were allowed access to buildings and pasture at all times but were kept in for two nights and days in the middle of the study period due to inclement weather and 4) the Free group were allowed free access to all pasture, cubicle housing, and the automated milking system (AMS) at all times during the study. Group location at pasture or in buildings was always governed by the farms, except in the case of the Free group, where cows had free access to all areas of the farm, and the Rotation 1b group where they had access to the daily selected pasture and housing. ‘Buildings’ networks for the Night-housed, Rotation 1b, Housed, and Free groups therefore represent milking times *and* being housed in buildings, whereas ‘Buildings’ networks for the Strip-grazed, Rotation 1a, Rotation 2, and the Stable groups are solely comprised of milking times (Table 1).

### 2.2 Equipment

Nylon cattle collars with a plastic clasp (Suevia Haiges, Germany) were fitted with a proximity device and a GPS receiver such that one device lay at either side of the animal’s neck. Positioning of the proximity sensors is challenging on large animals, due to shielding of radio signals and anisotropies caused by the body of the animal, but as we were primarily interested in the transmission of infection via the oro-nasal route, it made practical sense to attach the tag to a neck collar, to which most cows in the study were already accustomed. The GPS receivers (i-GotU GT-120 and GT-600 devices, Mobile Action Technology Inc., Taiwan) were configured to record fixes every ten minutes. The hardware of the proximity device is based on a design developed by the OpenBeacon project (http://www.openbeacon.org/). The proximity sensing platform has been designed by the SocioPatterns collaboration consortium (http://www.sociopatterns.org/), and has been used in contact studies of humans, sheep and dogs (Cattuto et al., 2010; Ozella et al., 2020; Wilson-Aggarwal et al., 2019). Sensors in close proximity exchange with one another a maximum of about 1 power packet per second, and the exchange of low-power radio-packets is used as a proxy for the spatial proximity of the animals wearing the sensors (Cattuto et al., 2010). Proximity relations between devices are assessed based on the difference between received and transmitted radio signal strengths. A contact event occurs if at least one data packet is exchanged between two devices during a continuous 20-second time window, and a contact is considered broken if a 20-second time window passes without data exchange (Cattuto et al., 2010; Kiti et al., 2019). Contact durations were therefore measured in 20-second blocks.

### 2.3 Validation in cattle

We assessed the performance of the proximity devices in two ways. Firstly, multiple tags were fixed to four wooden posts at different heights in a square pattern, posts were sequentially placed at increasing distances away from one another, with and without an obstruction between the tags. From this we determined that an attenuation threshold of -70 dBm was an appropriate proxy for contacts of cattle in the 1–1.5 m range, likely to be relevant for detecting potential transmission of pathogens that may be spread oro-nasally or by direct contact (Xie et al., 2007). Secondly, we used video observations of three cows interacting in a yard and confirmed that devices registered proximity between cows. In one case, packets were exchanged between devices that were >5 m apart, likely due to signal propagation on metal surfaces. However, this ‘false positive’ did not meet our criteria for a contact, and would therefore have been removed during data cleaning if this had occurred within the study data. The low-power radio frequency in use cannot propagate through the animal body and contacts are best recorded when the devices are face-to-face, which was confirmed by video analysis of cows wearing the devices standing parallel to one another at a feed trough. Some proximity devices (n = 16) recorded abnormally high contacts during very short timeframes (Fig. S1). If a proximity device recorded more contacts than 95 % of the total contacts recorded by all tags within a 30-minute time frame, we removed all data from that device, as we considered these contacts highly unlikely to be biologically feasible. Some devices recorded similar patterns of contact to others but with overall many fewer interactions. This may have been due to altered positioning of the tags on these animals, but as these data showed biologically plausible patterns of contacts, they were retained for the analysis.

Despite these limitations, the cleaned data are likely to provide a good approximation of proximity between cattle, as they have done in previous studies in similar, indoor barn environments (Milwid et al., 2019b).

### 2.4 Network and statistical analysis

#### 2.4.1 Do contact patterns vary through time and space?

We calculated the mean frequency of contacts between all group members for each group for the whole study period, and at two-hourly and daily intervals from 24:00 h to 24:00 h. Contacts from the whole study period were aggregated to form ‘Full networks’, where cows were represented as nodes, and contacts were represented as undirected edges. Each combination of cow pairs is referred to as a dyad. Edges were weighted by the total duration of contact (measured in 20-second units as per the definition of a contact) between the two cows for the relevant time period, at an appropriate scale (e.g. minutes-per-day on Full networks).

To explore differences between contacts (defined by proximity data) when cows were in buildings or out at pasture, we used the GPS data from each cow to classify the group location. Using open-source GIS software (QGIS Development team, 2019), areas of the farm were split into either i) ‘Pasture’, comprising all grassland available to cattle, or ii) ‘Buildings’, comprising any part of the farm where cattle were not on pasture, including the collecting yard, milking parlour, housing, loafing area, etc. For each 30 min window of the study period, we defined a cow’s individual location as where >50 % of their individual GPS fixes were recorded. Group location (Pasture or Buildings) was then defined as the area containing over 75 % of cows for the same 30-minute time window. Where less than 75 % of the group was in one location, we categorised this time as ‘Split’ (usually when the group was moving from one location to another). We aggregated the proximity data by summing the contact durations for contacts that occurred within the 30-minute windows for each respective location to create three spatial proximity networks, termed the ‘Buildings’, ‘Pasture’ and ‘Split’ networks, which span the entire study period. We compared the relative proportion of time spent in each spatial location with the frequency of contacts that occurred in each location. We tested correlation between these networks using a quadratic assignment procedure from the R package ‘sna’ (Butts, 2016). Due to the high density of the Full and Spatial networks, with most cows in contact at some point during the study period, we additionally assessed the networks based on stronger interactions by filtering out weaker edges. We removed edge weights below the 50th, 75th and 90th percentiles of edge weights in the unfiltered network, for example, F50 networks removed cow to cow relationships that were in the lowest 50th percentile of time spent together (James et al., 2009). We also tested correlations between Full and Spatial networks at each of these filtered levels (F50, F75, F90).

#### 2.4.2 Do contact frequency and duration vary among cows?

We assessed the centrality of cows within each group by calculating node degree (the total number of in-contact cows) and strength (the total amount of time each cow spent with any other cow) for each cow. Heterogeneity in contact rate is well-known to affect disease transmission (Lloyd-Smith et al., 2005; VanderWaal and Ezenwa, 2016), and can be assessed by the coefficient of variation (CV), which is calculated by dividing the standard deviation by the mean (May, 2006). CVs were calculated for degree (CV_degree_) and strength (CV_strength_) on the Full network and Buildings and Pasture networks for each group. In general, higher CV values indicate increasing variation between values and therefore, a CV_degree_ that tends towards zero indicates a more homogeneously mixing population (May, 2006).

Measures of the network position for different individuals are inherently non-independent and thus violate the assumptions of many conventional statistical techniques. Therefore, to conduct statistical inference we constructed null, randomised networks to test if values from our observed networks were statistically significantly different from what would be expected by chance (Croft et al., 2011). We randomised static networks by first creating 4999 new Erdӧs-Rényi graphs (Erdös and Rényi, 1959) with the same number of edges and nodes as the original network and then randomly allocating edge weights from the observed network to the new edges in the Erdӧs-Rényi network. Metrics were considered statistically significantly different from random if their values lay below 2.5 % or above 97.5 % of the 4999 randomised values.

#### 2.4.3 Do cows exhibit social preferences and are they consistent over time?

To see if cows preferentially spent more time with particular other cows, for every individual we measured the CV for the total contact times that each focal cow spent with others (CV_cddyad_). We generated 4999 permuted networks that randomised dyadic contact durations. First, we took the raw list of contacts (the edgelist) and then randomly re-allocated contact durations among individual contacts to create a random edgelist. Second, the random edgelist was then aggregated by summing the weights from individual contacts for each dyad. Third, we created a new unweighted Erdӧs-Rényi network with the same number of nodes and edges as the random edgelist. Finally, the aggregated edge weights from the random edgelist were randomly allocated to new edges in the Erdӧs-Rényi network, thereby keeping the overall amount of time cattle spent together the same as the observed network, but changing how this was distributed among animals. We tested the hypothesis that the CV_cddyad_ values of cows in observed networks were different to the CV_cddyad_ values of cows in randomised networks on the basis that if the focal cow spent disproportionately long periods of time with particular other cows, it might have a more variable range of contact durations. We calculated a two-tailed empirical *P* value by dividing the number of times the observed CV_cddyad_ was outside the central 95 % of randomised CV_cddyad_ values by the number of CV_cddyad_ vaues from random networks plus the observed CV_cddyad_ value (4999 + 1) as it might have feasibly come from a random distribution (Boyland et al., 2016).

To assess the tendency of cows to repeatedly interact with the same cows over time, we measured the temporal variation in contact between each dyad. In each two-hour block of the study period, we attributed a 1 if a dyad was observed to have contact and a 0 if there was no contact during that time. Null models (n = 4999) were generated by randomly distributing the 1 s and 0 s within each two-hour block among all dyads. We calculated the proportion of two-hour blocks in which cows interacted per 24 h, e.g. 6/12 = 0.5. We then calculated the CV of the proportions from each study day (CV_proportion_) for each dyad, where a low CV_proportion_ indicates little variation in the amount of time they spent together between each study day and a high CV_proportion_ indicates cows spending highly variable amounts of time with one another. We removed dyads with no connections at all from the analysis to focus on the variation in contacts rather than non-contacts. We tested the hypothesis that the interactions between dyads in the observed networks were different to those of dyads in random networks and calculated empirical *P* values by the same method as previously described.

#### 2.4.4 Do cows form discrete communities and are they consistent over time?

We performed community detection on unfiltered, unweighted Full and Spatial (Buildings, Pasture and Split) networks using the fast greedy algorithm (Clauset et al., 2004), as implemented in ‘igraph’ (Csardi and Nepusz, 2006). Modularity is a measure of how divided the communities are within a network, as it is related to the number of nodes, and our networks are of different sizes, we used a relative measure, as outlined by Sah et al. (2017), where we calculated the maximum modularity that could be achieved with a network of that size (*Q_max_;* Sah et al., 2017) and the modularity of the observed network (*Q*), and then calculated the relative modularity (*Q_rel_*) by dividing *Q* by *Q_max_*. Due to the high density of the Full and Spatial networks, we also performed the community detection and modularity analysis on these networks filtered by edge weights below the 50th (F50), 75th (F75), and 90th (F90) percentiles of contacts.

**Fig. 1:**
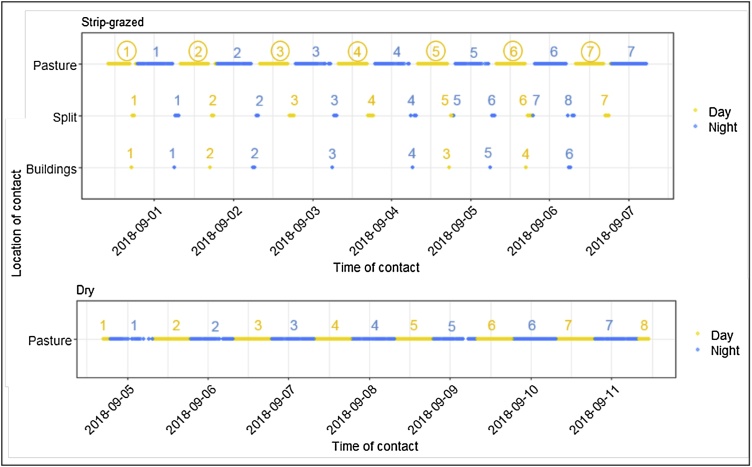
Examples of how contacts were grouped by different locations and times of day (i.e. Pasture, Buildings, and Split networks by day or night) to analyse the repeatability of dyads being in the same community over time. Each number represents a separate network formed from the contacts below it (represented by multiple overlapping points), the location is denoted by the y-axis and the colour represents the time of day (yellow = day, blue = night), where day is defined as 07:00–19:00. For example, the yellow ‘1’ in the top left of the plot represents the first Pasture, day network for the Strip-grazed group. We calculated communities in each of these networks, noted for each dyad if they were in the same community in each network and then tested the repeatability of dyads being in the same community across the sequential networks for each network category, (e.g. the pasture day category compared yellow 1, 2, 3, 4, 5, 6, 7 in the Pasture group – circled in yellow on schematic). As the Dry group stayed in one location for the majority of the study, the networks were only divided by time of day, not location.

To assess if the composition of communities were repeatable in time and space, we extracted the contacts by their location classification (Buildings, Pasture, and Split). We then grouped contacts that occurred during a similar time period, divided by a change in location or a change from day (defined as 07:00–19:00) to night, into several spatio-temporal networks named by their location (Pasture, Buildings, Split) and then the time of day (day or night), for example ‘Pasture day’ or ‘Pasture night’ (Fig. 1). If groups had less than three networks within a certain category, they were removed from the analysis. For each time period we created a weighted network with aggregated contacts and calculated communities using the fast-greedy algorithm. For each dyad in each network, we assigned 1 if both cows were in the same network and 0 if they were in a different network. To assess statistical significance, at this point we randomly distributed the 1 s and 0 s among dyads (n = 4999) and then proceeded with the same analysis for randomised and observed data. We calculated the ‘repeatability’ (estimated from the variance component of a generalised linear mixed model using a binomial error structure in the package ‘rptR’ (Stoffel et al., 2017)) to determine the stability of dyads being in the same community in multiple networks over time. Repeatability values can range from 0, indicating a lack of stability in community membership, to 1, indicating perfectly stable community membership.

All data analysis and manipulation was performed in R version 3.5.3 (R Core Team, 2019) unless otherwise stated. Networks were constructed and network measures (degree, strength, community detection and modularity) were calculated in the R package “igraph” (Csardi and Nepusz, 2006).

All work with animals was approved by the University of Exeter College of Life and Environmental Sciences (Penryn Campus) ethics committee (eCORN000087 v4.6).

## 3 Results

Mean group size at the start of each deployment was 85 (range 33–175). All cows in each group on the day of deployment were collared, 14 collars in total fell off during the study periods, with no more than 3 collars falling off on one farm. Data from cows that were not present in the group for the entire study period or for which we had incomplete data were removed from our analyses. Complete proximity data were available for a total of 585 out of 777 collared cows and therefore the number of animals in our networks is lower than the group size and ranged from 22 to 100 (mean = 65 cows, standard deviation (SD) = 28 cows; Table 1). The mean duration of time cows spent in recorded proximity to other cows over all groups was 27 min per day (SD =11 min, Table S1). Mean contact rate over the study period among groups was 0.20 contacts per hour per dyad (SD = 0.07) with the Free group and the Stable group having the highest and lowest mean contact rates respectively (Table S1). Edge densities of all Full networks were very high, although never fully saturated, such that all cows did not come into the proximity of all other cows in their groups (mean edge density among groups = 0.95, SD = 0.03, Table S1).

### 3.1 Do contact patterns vary through time and space?

**Fig. 2:**
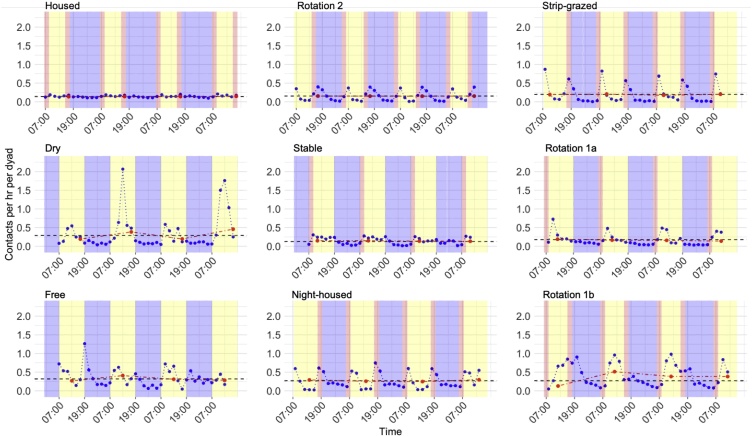
Temporal variation in numbers of contacts recorded between cows in nine groups of dairy cattle. Contacts during the day (yellow shading) and night (blue shading) were recorded by proximity loggers and contact frequency is averaged per hour per dyad (cow-cow pair) and aggregated into varying time periods; the whole study period (mean = 7 days; black solid line), daily (red circles and dashed lines), and 2-hourly (blue circles and dotted lines). Daily and 2-hourly values are selected from the middle four days of the study period for brevity. Milking regimes (light red shading) varied by group and timings plotted here are those reported by farmers. There is little variation between days but strong within-day patterns are evident in most farms, with higher contacts around dawn and dusk, frequently aligned with milking time.

There was substantial temporal variation in contact frequency in most groups, when data were aggregated into 2-hourly windows (Fig. 2). Frequency was greatest when coinciding with management practices such as milking, which tended to occur early morning and early evening, or feeding, which tended to occur after milking. Although cows were not specifically gathered for milking or feeding in the Free group, there were still increases in contacts around dawn and dusk. Patterns of contact frequencies typically followed 24 h (e.g. Fig. 2, Rotation 1a and 1b) or 12 h cycles (e.g. Fig. 2, Strip-grazed, Rotation 2, Stable), thus the number of contacts aggregated over 24 h periods was consistent between days. Mean daily contact frequencies were similar to mean values calculated from contacts during the entire study period (Fig. 2). Contact frequencies over 2-hourly periods in the Housed and Stable groups showed far less temporal variation relative to other groups (Fig. 2). In groups with access to pasture, contact rates were close to zero at some points in the study, more often during the night. In contrast, contact rates never fell this low when groups were housed (Fig. 2).

**Fig. 3:**
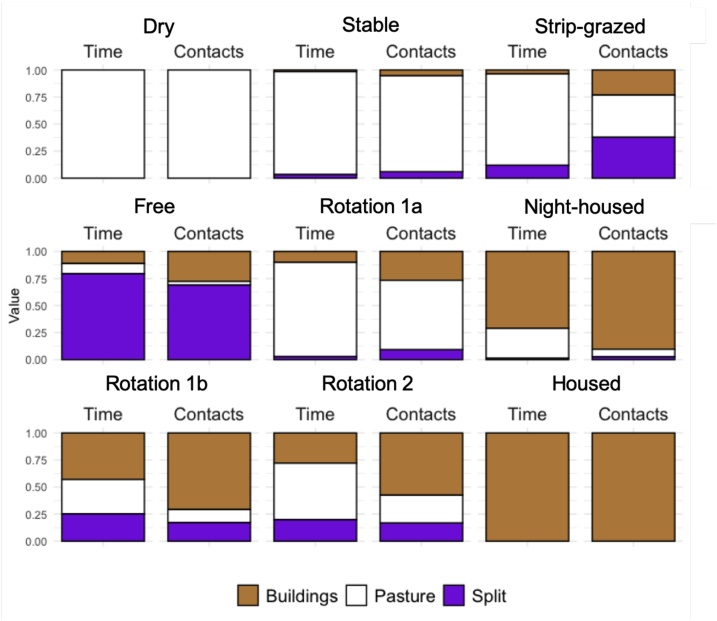
Relative amount of time and number of between-cow contacts in different farm areas recorded in seven groups of dairy cattle. Bar charts show the relative proportion of time and contacts recorded by proximity loggers in Buildings (brown) and at Pasture (white). Groups are defined as ‘Split’ when less than 75 % of cows are in either Buildings or Pasture (purple). Most cattle only had access to buildings or pasture at specified times, however, the Free group was allowed access to all farm areas and elected to spend the majority of time split into two groups.

**Fig. 4:**
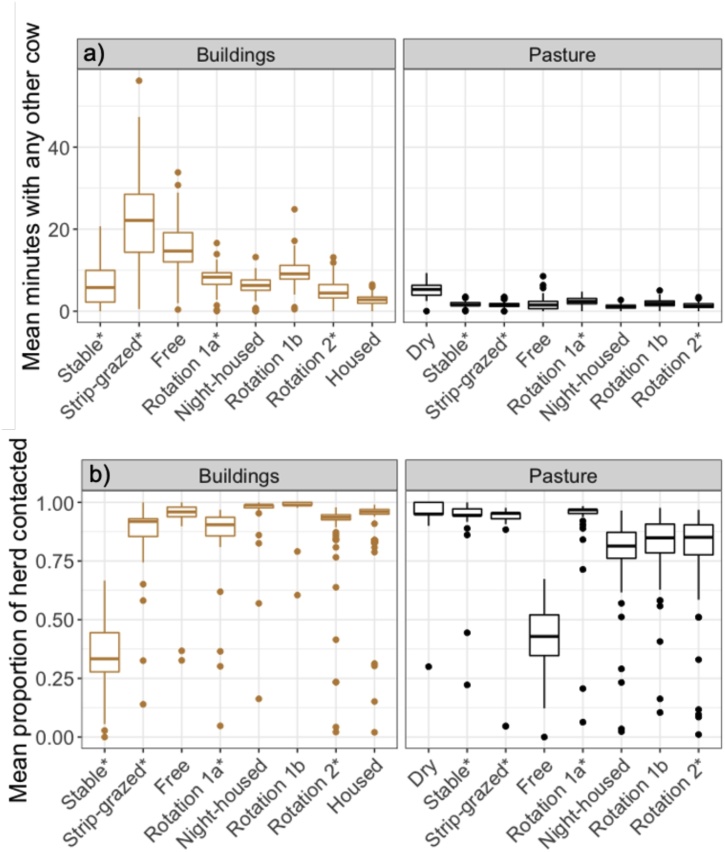
Distributions of the time cows spent together and the numbers of cows contacted in nine groups of dairy cattle at pasture and in buildings. a) Mean amount of time cows spent with other cows. b) Proportion of cows in the group with which they came into contact during the whole study period. Values are calculated for Pasture networks (black) and Buildings networks (brown). Groups are ordered by ascending group size from left to right and * denotes groups only in buildings for milking. Boxplots show the median, 25th and 75th percentiles of values and the upper and lower whiskers extend to the largest or smallest value no further than 1.5 times the interquartile range, data beyond this range are plotted as outlying points. In all comparable groups, less time is spent in proximity at pasture and on most farms, fewer cows are in proximity at pasture compared to in buildings. Housed and dry groups were only in buildings and at pasture respectively, therefore only appear on the corresponding plot.

Spatial analysis revealed substantial differences in contacts between locations. More contacts occurred in Buildings networks, compared to Pasture and Split networks, relative to the amount of time spent in these areas (Fig. 3). On average, cows had longer contact durations in Buildings than at Pasture (Fig. 4). For groups housed in buildings (Night-housed, Rotation 1b), and the Free and Rotation 2 groups, most cows contacted almost all other cows in the Buildings networks, and contacted fewer cows in Pasture networks (Fig. 4). However, where cattle were only brought into buildings for milking, most cows contacted fewer cows in Buildings networks (Stable, Rotation 1a), or showed no difference in the proportion of cows contacted (Strip-grazed) compared to Pasture networks. When cows had access to all areas of the farm, the group was most often split between buildings and grazing (Fig. 3).

Edge densities varied among the three spatial networks, with Buildings networks (mean edge density among groups = 0.85, SD = 0.20) and Pasture networks (mean = 0.82, SD = 0.17) denser than the Split networks (mean = 0.73, SD = 0.20). In the 7 groups with comparable networks, the quadratic assignment procedure demonstrated that daily Spatial networks were generally not correlated with one another (Table S2), except for the unfiltered Buildings and Split networks for the Strip-grazed group (*r* = 0.3, *P* < 0.001, Table S2). Filtering networks to retain only the strongest contacts did not noticeably affect correlation scores (Table S2).

### 3.2 Do contact frequency and duration vary among cows?

**Fig. 5:**
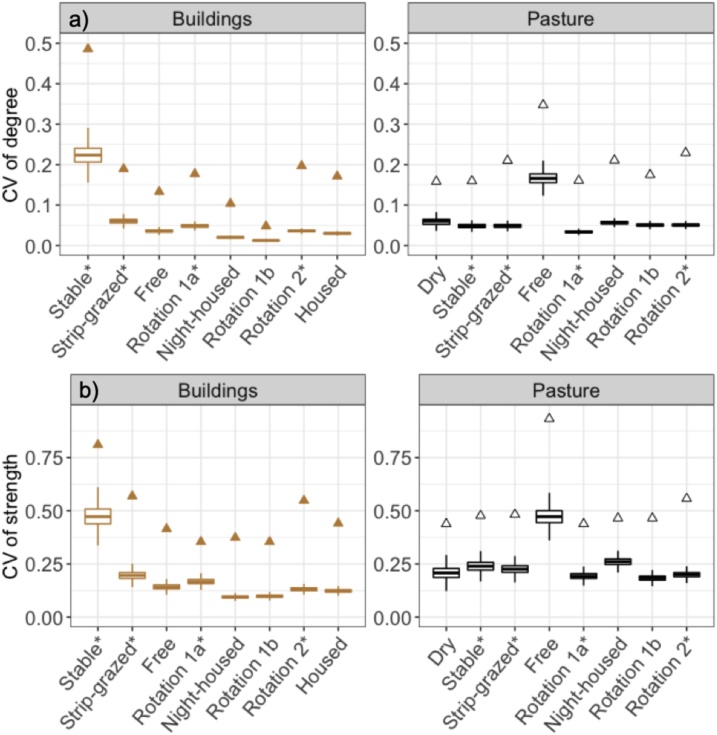
Variation between cows in the number and duration of their contacts (triangles) compared to variation in random networks in nine groups of dairy cattle at pasture and in buildings. a) Coefficient of Variation (CV) of degree showing variation in number of other cows each cow contacted b) CV of strength showing variation in duration of contact between-cows. Triangles indicate observed values above the upper 95 % bound of random network values calculated for contacts on pasture (‘Pasture network’; black) and in buildings (‘Buildings network’; brown). Boxplots represent the distribution of CV values calculated from randomised networks (n = 4999). Median, 25th and 75th percentiles of values are shown and the upper and lower whiskers extend to the largest or smallest value no further than 1.5 times the interquartile range, data beyond this range are not plotted. Groups are ordered by ascending group size from left to right and * denotes groups only in housing for milking. Housed and dry groups were only in buildings and at pasture respectively, therefore only appear on the corresponding plot.

Variation among cows in their number of contacts (CV_degree_) and their total contact duration with other cows (CV_strength_) was higher in observed than in randomised networks (Fig. 5). There was consistently greater heterogeneity in the duration of contacts (strength) than the number of contacts (degree) within groups (Fig. 5), likely due to dense networks limiting the extent of variation possible for degree. Generally, greater variation in degree and strength was found in Pasture networks, and this was most marked in the Free group where CV values on Pasture networks were more than double those found in Buildings networks (Fig. 5). However, groups that were only inside for milking occasionally had greater variation in buildings; in degree for the Stable and Rotation 1a groups and in strength for the Stable and Strip-grazed groups (Fig. 5), likely related to the particular system of milking on those farms. Differences in individual variation between farms was not clearly associated with group size (Fig. 5).

### 3.3 Do cows exhibit social preferences and are they consistent over time?

**Fig. 6:**
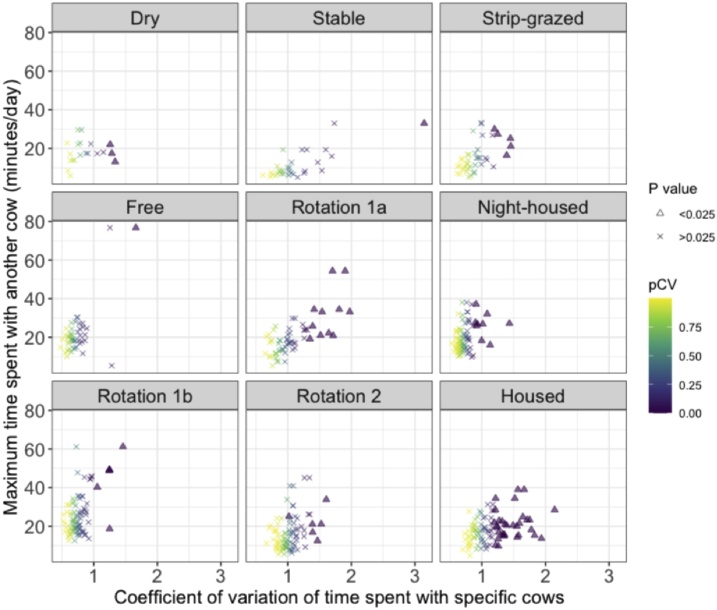
Social preference based on cow-cow interactions in nine groups of dairy cattle. Plots show the variation in time spent with other cows (CV_cddyad_), compared with the maximum time a cow spent with a single group member. Each cow is represented by a point, coloured by the P value for the coefficient of variation (CV) compared to CV values calculated on random networks (P values < 0.025 are shown as triangles and > 0.025 are shown as crosses). A small number of cows in each group show preference for spending more time with particular other cows, however, the variation in the interactions of most cows did not differ significantly from random. To aid visualisation of more sociable cows, 37 cows with mean contact time with other cows below one minute were removed from this plot.

Some cows in each group showed greater variation in the duration of time they spent with individual cows, when compared to randomised networks, suggesting social preference (Fig. 6). The mean proportion of cows that exhibited greater variation than random and spent a substantial amount of time (over 21 min per day) in recorded contact with their closest contact was 7.5 % among groups (SD = 5.1 %, range = 2.0–16.0 %). The majority of cows in each deployment spent similar amounts of time in proximity to all other individuals in the group, akin to what might be expected at random (Fig. 6).

A small proportion of dyads in each group (mean = 3.7 %, SD = 1.5 %, range = 2.1–5.2 %) spent more consistent amounts of time together from day to day than would be expected if contacts were assorted at random (*P* < 0.025; Fig. S2). On average these ‘consistent dyads’ spent a mean time of 30 min (SD = 11 min, interquartile range (IQR) = 25–38 min) together per 2 h window, compared to a mean of 18 min (SD = 10 min, IQR = 12–25 min) in the rest of the dyads. Over all groups, 14 dyads were recorded spending a mean of over 60 min with each other out of every 120 min.

### 3.4 Do cows form discrete communities and are they consistent over time?

Few communities were found in the denser, unfiltered, unweighted networks (Fig. S3). These networks also tended to have very low relative modularity values (*Q_rel_* < 0.03, Table S3 and Fig. S5). As expected, when networks were filtered and weaker edges were removed, more communities were detected. The largest numbers of communities (≥ 20) were found in F90 networks in the Strip-grazed group Split network and the Free group Pasture network (Fig. S3), however the relative modularity of these networks did not differ from random (Fig. S5). Observed modularity was rarely substantially greater than that of random networks (Table S3 and Fig. S5), and the lack of a consistent pattern of statistically significant results suggests the few cases where observed values differed statistically significantly from random were caused principally by multiple testing.

Although the consistency of our observed communities was statistically significantly greater than expected at random, the absolute observed repeatability values were still very low, indicating that the communities we detected in the temporal networks did not consistently contain the same cows (Fig. S4). The exception to this finding was the Pasture day networks of the Stable group, which were far more consistent than the night networks, or any other categories of the spatio-temporal networks (day repeatability = 0.22, night repeatability = 0.05; Fig. S4).

## 4 Discussion

Against a background of very dense, highly connected networks when viewed as a whole, our finer-scale spatio-temporal analyses show evidence of temporal variation in contact frequency among cows, differing contact patterns between locations within the farm, and evidence of stronger bonds between only a small subset of animals.

Contacts lasted longer in buildings compared to those at pasture, reflecting the evident space restrictions in bringing cattle and their resources closer together, but perhaps also suggesting that cattle will spend less time very close together where the distribution of resources permits such behaviour. For most deployments, cow contacts were proportionally higher in buildings, suggesting, as might be expected, that direct transmission of infections is likely to be increased when animals are housed indoors, at least at the stocking densities present in our study farms. Contacts may have been underestimated when sensors were facing opposite directions, particularly in indoor housing, where cows are frequently positioned with their heads side by side due to feeding along a barrier or lying in cubicles. Therefore, the comparably higher contact frequencies and durations detected in Buildings compared to Pasture may actually be greater than we observed. In three groups that were in buildings only for milking, the mean number of individuals contacted in buildings was less than at pasture. We suggest this was likely due to the milking routine on these farms, whereby cattle were walked into a collecting yard, before entering the parlour in small groups and after milking were allowed to walk back to pasture in their own time. Contrary to intuitive assumptions that milking time tightly groups animals together (Barlow et al., 1997; Bekara et al., 2014), we have found that, depending on the routine of the farm, it might represent a time where cattle are more clearly divided into separate clusters. Despite cows in the Free group being milked individually on the automatic milking system, we recorded peaks in the frequency of contacts around dawn and dusk which may reflect a historical pattern of grouping together at milking (as the farm introduced the automated milking system only in the previous year), feeding around these times, or could suggest that there are times of day, regardless of management and weather conditions, where cows are naturally more interactive (Stoye et al., 2012). The networks formed by the Strip-grazed group during the transition to milking (Split networks) and during milking (Buildings networks) showed more similarity than seen in other groups which, although still relatively low (r = 0.3; Table S2), could reflect a tendency for these cows to travel to and from milking in a similar order, as well as entering the parlour in a similar order (Beggs et al., 2018; Bouissou et al., 2001; Mcvey et al., 2020).

We predicted that more consistent network communities might form, and modularity might be higher at pasture, as cows would have the opportunity to space out and show social preferences. Indeed, when cattle were at Pasture there were occasional two-hour periods with hardly any contacts at all, particularly at night, perhaps indicating a preference for cattle to be further apart at times when there is less competition for resources. On the farm where cattle were free to roam wherever they chose (Free group), many more network communities were detected and there was greater variation in the number and duration of contacts among individuals in the Pasture networks. The limitations of one field, or part of a field, available to strip-grazing and rotational-grazing groups might mean that cattle tend to remain more clustered together as one unit, as reported in contact networks of sheep at different stocking densities (Ozella et al., 2020), whereas the availability of more extensive grazing (as was the case for the Free group in our study) evidently facilitates formation of sub-groups. Generally, there was no consistent difference between the number of communities formed, or the modularity of the Pasture networks, compared to the Buildings networks. This may be in part due to difficulties with community detection methods in networks as dense as in our study (Clauset et al., 2004), and the resolution limit problem, where smaller communities may fail to be detected in some networks (Chen et al., 2018) making the comparison of modularity on networks with differing numbers of edges complex.

Cattle are reported to form strong social bonds (Bouissou et al., 2001; Duncan et al., 2012; Handcock et al., 2009) and we found evidence of consistent and extended durations of close contact indicative of social preferences, but between only a small proportion of individuals in each group. Overall, we found that most cattle in the sampled groups spent time in proximity to one another in a manner similar to random assortment (Mcvey et al., 2020). This may be reflective of dynamic groups in most of the farms we studied, as even the ‘stable’ group that were kept as a single herd, were sourced from different farms and had not been reared together, in contrast to many groups in beef herds. This suggests that relatively fewer strong social bonds are formed in the herds in our study might be expected in a less managed environment (Bouissou et al., 2001; McLennan, 2013). However, we acknowledge our study periods were relatively short, and focal studies over longer time periods may reveal more complex and/or sustained social relationships among cows (Rocha et al., 2020).

Despite collaring all cows that were present on the start date of each deployment, we were unable to obtain data from some devices (mean of all groups = 23 %; Table 1). A small proportion of these missing data was due to collars coming off (i.e. found on floor by farmer; n = 15), and we cannot rule out that this was because of particular behaviours shown by those cows that might cause the collar to have been removed, although this was not reported by farmers. However, the major reason for a lack of data was software failure. Therefore, we believe that the sampled individuals in this study were not likely to be biased towards a particular type of cow, e.g. those exhibiting or being subject to more aggressive encounters resulting in physical damage to the proximity device, rather the missing data occurred via a random process of technical malfunction. However, missing nodes may have reduced the study’s ability to detect dyadic relationships (VanderWaal et al., 2016). Repeated sampling of individuals with the same and different groups could allow a more robust analysis of this parameter in future studies.

Contact structure among hosts can alter pathogen transmission dynamics (Bansal et al., 2007). Modelling studies have shown that modularity values of more than 0.45–0.6 may reduce the peak but prolong the duration of an outbreak via increased transmission within groups and decreased transmission between groups (Rozins et al., 2018; Sah et al., 2017). Only highly filtered networks of the Free and Stable groups in the present study had modularity values different from random that might be substantial enough to potentially impede disease spread, and it is more likely that the weak and temporally unstable community structure in our networks would facilitate rather than impede, the spread of highly-transmissible infections. Again, the exception was the Free group, in which individuals were free to aggregate of otherwise as they chose. In this group, for large proportions of the study period, the cows were split between the pasture and the buildings, which might impede the spread of disease throughout the herd. The differences seen in this group suggest that, in addition to the reported welfare benefits (Edgar et al., 2013; Mee and Boyle, 2020), cattle that are allowed free access to all areas of the farm may form social structures with divisions that hamper the spread of infections. Though our study only represents contact patterns over a period of good weather in summer and autumn, if given the choice in poor weather conditions, cattle may favour indoor spaces (Charlton et al., 2011), where transmission of infections might be enhanced.

When daily networks were calculated, contact frequency was similar between days for most groups, yet when contacts from the present study were aggregated by shorter time periods (2 h), variation in contact frequency became much more apparent. Modelling of disease transmission on high-temporal-resolution networks (Chen et al., 2014) has highlighted how aggregation of contacts at the hourly or 2-hourly level can be associated with higher variability in epidemic size, with lower values of R_0_ (between 1 and 2) compared to aggregating over longer time-periods (Chen et al., 2014). The time-scale over which contacts are aggregated should also be guided by the biology of the pathogen of interest (Dawson et al., 2019); data aggregated over short time windows are more likely to be informative for modelling the transmission of infections with short infectious periods (Perkins et al., 2009). Yet for chronic infections with longer infectious periods, it is likely that incorporating such a high resolution of contacts in transmission models will have less impact on transmission dynamics, while cumulative values of contact duration or strength of contacts may remain important (Read et al., 2008). Heterogeneity in contact rate and strength can affect the speed and extent of pathogen transmission through a population (May, 2006). Despite cows being grouped closely together at many points during the study period, they showed substantial differences in their sociality such that incorporating the contact structure of cattle into modelling studies is likely to improve model predictions, even at coarse temporal aggregations.

## 5 Conclusions

The changing face of the dairy industry in the United Kingdom over recent years has resulted in many herds increasing in size (AHDB Dairy, 2019) and changes to how cattle are managed, with a trend towards more cattle being kept indoors for more of the year, or, in some cases, entirely in indoor units (Haskell et al., 2006). The increased contact among cattle in buildings demonstrated in this study suggests this trend could increase the risks of transmission of infections (Arnott et al., 2016). In order to mitigate these effects, consideration might be given to housing design in order to allow sufficient space for cattle to disperse and to reduce competition for resources within small areas. Milking routine is often expected to increase contact between cattle, although we found that this is not inevitable, and some milking protocols might create relatively fewer opportunities for disease transmission. Dairy cow social networks have typically been described as a single, unstructured groups; indeed, our study networks are densely connected during weekly and daily aggregations. However, when the network is divided in space and time, there are differences in cattle interactions that are not apparent at larger scales. From studying multiple groups of cattle, we have found that differences in management, even among dairy herds within the same region, can influence social structure which might therefore have implications for disease transmission. Such variation should be considered when parameterising mathematical and statistical models of disease spread in livestock.

## Author contributions

HRF and RAM conceived the study. HRF collected the data, LO and JWA provided technological support. LO, LG and CC processed the proximity sensor data. MJS and TJM assisted with data analysis. HRF wrote the manuscript draft. All authors reviewed and approved the final manuscript.

## Funding

This work was supported by an iCASE studentship from the 10.13039/501100000268BBSRC (BB/M015874/1) in partnership with the Animal and Plant Health Agency. LO, LG, CC acknowledge partial support from the Lagrange Project of ISI Foundation funded by CRT Foundation.

## Declaration of Competing Interest

None.

## References

[bib0005] AHDB Dairy (2019). Agriculture and Horticulture Development Board.

[bib0010] Álvarez J., Bezos J., de la Cruz M.L., Casal C., Romero B., Domínguez L., de Juan L., Pérez A. (2014). Bovine tuberculosis: Within-herd transmission models to support and direct the decision-making process. Res. Vet. Sci..

[bib0015] Anderson R.M., May R.M. (1992).

[bib0020] Arnott G., Ferris C.P., O’Connell N.E. (2016). Review: welfare of dairy cows in continuously housed and pasture-based production systems. Animal.

[bib0025] Bansal S., Grenfell B.T., Meyers L.A. (2007). When individual behaviour matters: homogeneous and network models in epidemiology. J. R. Soc. Interface.

[bib0030] Bansal S., Read J., Pourbohloul B., Meyers L.A. (2010). The dynamic nature of contact networks in infectious disease epidemiology. J. Biol. Dyn..

[bib0035] Barlow N.D., Kean J.M., Hickling G., Livingstone P.G., Robson A.B. (1997). A simulation model for the spread of bovine tuberculosis within New Zealand cattle herds. Prev. Vet. Med..

[bib0040] Beggs D.S., Jongman E.C., Hemsworth P.H., Fisher A.D. (2018). Short communication: milking order consistency of dairy cows in large Australian herds. J. Dairy Sci..

[bib0045] Bekara M.E.A., Courcoul A., Bénet J.J., Durand B. (2014). Modeling tuberculosis dynamics, detection and control in cattle herds. PLoS One.

[bib0050] Bouissou M., Boissy A., Le Neindre P., Veissier I. (2001). Social Behaviour in Farm Animals.

[bib0055] Boyland N.K., Mlynski D.T., James R., Brent L.J.N., Croft D.P. (2016). The social network structure of a dynamic group of dairy cows: from individual to group level patterns. Appl. Anim. Behav. Sci..

[bib0060] Brooks Pollock E., Keeling M.J. (2009). Herd size and bovine tuberculosis persistence in cattle farms in Great Britain. Prev. Vet. Med..

[bib0065] Butts C.T. (2016). sna: Tools for social network analysis. R package version.

[bib0070] Cattuto C., van den Broeck W., Barrat A., Colizza V., Pinton J.F., Vespignani A. (2010). Dynamics of person-to-person interactions from distributed RFID sensor networks. PLoS One.

[bib0075] Charlton G.L., Rutter S.M., East M., Sinclair L.A. (2011). Preference of dairy cows: indoor cubicle housing with access to a total mixed ration vs. Access to pasture. Appl. Anim. Behav. Sci..

[bib0080] Chen S., White B.J., Sanderson M.W., Amrine D.E., Ilany A., Lanzas C. (2014). Highly dynamic animal contact network and implications on disease transmission. Sci. Rep..

[bib0085] Chen S., Ilany A., White B.J., Sanderson M.W., Lanzas C. (2015). Spatial-temporal dynamics of high-resolution animal networks: What can we learn from domestic animals?. PLoS One.

[bib0090] Chen T., Singh P., Bassler K.E. (2018). Network community detection using modularity density measures. J. Stat. Mech. Theory Exp..

[bib0095] Clauset A., Newman M.E.J., Moore C. (2004). Finding community structure in very large networks. Phys. Rev..

[bib0100] Conlan A.J.K., McKinley T.J., Karolemeas K., Brooks Pollock E., Goodchild A.V., Mitchell A.P., Birch C.P.D., Clifton-Hadley R.S., Wood J.L.N. (2012). Estimating the hidden burden of bovine tuberculosis in Great Britain. PLoS Comput. Biol..

[bib0105] Courcoul A., Ezanno P. (2010). Modelling the spread of Bovine Viral Diarrhoea Virus (BVDV) in a managed metapopulation of cattle herds. Vet. Microbiol..

[bib0110] Craft M.E. (2015). Infectious disease transmission and contact networks in wildlife and livestock. Philos. Trans. Biol. Sci..

[bib0115] Croft D.P., Madden J.R., Franks D.W., James R. (2011). Hypothesis testing in animal social networks. Trends Ecol. Evol..

[bib0120] Crump A., Jenkins K., Bethell E.J., Ferris C.P., Arnott G. (2019). Pasture access affects behavioral indicators of wellbeing in dairy cows. Animals.

[bib0125] Csardi G., Nepusz T. (2006).

[bib0130] Dawson D.E., Farthing T.S., Sanderson M.W., Lanzas C. (2019). Transmission on empirical dynamic contact networks is influenced by data processing decisions. Epidemics.

[bib0135] Duncan A.J., Gunn G.J., Lewis F.I., Umstatter C., Humphry R.W. (2012). The influence of empirical contact networks on modelling diseases in cattle. Epidemics.

[bib0140] Edgar J.L., Mullan S.M., Pritchard J.C., McFarlane U.J.C., Main D.C.J. (2013). Towards a “good life” for farm animals: development of a resource tier framework to achieve positive welfare for laying hens. Animals.

[bib0145] Erdös P., Rényi A. (1959). On random graphs. Publ. Math..

[bib0150] Foris B., Zebunke M., Langbein J., Melzer N. (2018). Comprehensive analysis of affiliative and agonistic social networks in lactating dairy cattle groups. Appl. Anim. Behav. Sci..

[bib0155] Gygax L., Neisen G., Wechsler B. (2010). Socio-spatial relationships in dairy cows. Ethology.

[bib0160] Handcock R.N., Swain D.L., Bishop-Hurley G.J., Patison K.P., Wark T., Valencia P., Corke P., O’Neill C.J. (2009). Monitoring animal behaviour and environmental interactions using wireless sensor networks, GPS collars and satellite remote sensing. Sensors.

[bib0165] Haskell M.J., Rennie L.J., Bowell V.A., Bell M.J., Lawrence A.B. (2006). Housing system, milk production, and zero-grazing effects on lameness and leg injury in dairy cows. J. Dairy Sci..

[bib0170] James R., Croft D.P., Krause J. (2009). Potential banana skins in animal social network analysis. Behav. Ecol. Sociobiol..

[bib0175] Kiti M.C., Melegaro A., Cattuto C., Nokes D.J. (2019). Study design and protocol for investigating social network patterns in rural and urban schools and households in a coastal setting in Kenya using wearable proximity sensors [version 2; peer review: 2 approved]. Wellcome Open Res..

[bib0180] Lloyd-Smith J.O., Schreiber S.J., Kopp P.E., Getz W.M. (2005). Superspreading and the effect of individual variation on disease emergence. Nature.

[bib0185] Marcé C., Ezanno P., Seegers H., Pfeiffer D.U., Fourichon C. (2011). Within-herd contact structure and transmission of *Mycobacterium avium* subspecies *paratuberculosis* in a persistently infected dairy cattle herd. Prev. Vet. Med..

[bib0190] May R.M. (2006). Network structure and the biology of populations. Trends Ecol. Evol..

[bib0195] McLennan K.M. (2013).

[bib0200] Mcvey C., Hsieh F., Manriquez D., Pinedo P. (2020). Mind the queue : a case study in visualizing heterogeneous behavioral patterns in livestock sensor data using unsupervised machine learning techniques. Front. Vet. Sci..

[bib0205] Mee J.F., Boyle L.A. (2020). Assessing whether dairy cow welfare is “better” in pasture-based than in confinement-based management systems. N. Z. Vet. J..

[bib0210] Milwid R.M., O’Sullivan T.L., Poljak Z., Laskowski M., Greer A.L. (2019). Comparing the effects of non-homogenous mixing patterns on epidemiological outcomes in equine populations: a mathematical modelling study. Sci. Rep..

[bib0215] Milwid R.M., O’Sullivan T.L., Poljak Z., Laskowski M., Greer A.L. (2019). Validation of modified radio-frequency identification tag firmware, using an equine population case study. PLoS One.

[bib0220] Ozella L., Langford J., Gauvin L., Price E., Cattuto C., Croft D.P. (2020). The effect of age, environment and management on social contact patterns in sheep. Appl. Anim. Behav. Sci..

[bib0225] Perkins S.E., Cagnacci F., Stradiotto A., Arnoldi D., Hudson P.J. (2009). Comparison of social networks derived from ecological data: implications for inferring infectious disease dynamics. J. Anim. Ecol..

[bib0230] QGIS Development team (2019).

[bib0235] R Core Team (2019).

[bib0240] Read J.M., Eames K.T.D., Edmunds W.J. (2008). Dynamic social networks and the implications for the spread of infectious disease. J. R. Soc. Interface.

[bib0245] Rocha L.E.C., Terenius O., Veissier I., Meunier B., Nielsen P.P. (2020). Persistence of sociality in group dynamics of dairy cattle. Appl. Anim. Behav. Sci..

[bib0250] Rozins C., Silk M.J., Croft D.P., Delahay R.J., Hodgson D.J., McDonald R.A., Weber N., Boots M. (2018). Social structure contains epidemics and regulates individual roles in disease transmission in a group-living mammal. Ecol. Evol..

[bib0255] Sah P., Leu S.T., Cross P.C., Hudson P.J., Bansal S. (2017). Unraveling the disease consequences and mechanisms of modular structure in animal social networks. Proc. Natl. Acad. Sci. U. S. A..

[bib0260] Silk M.J., Croft D.P., Delahay R.J., Hodgson D.J., Weber N., Boots M., McDonald R.A. (2017). The application of statistical network models in disease research. Methods Ecol. Evol..

[bib0265] Springer A., Kappeler P.M., Nunn C.L. (2017). Dynamic vs. Static social networks in models of parasite transmission: predicting Cryptosporidium spread in wild lemurs. J. Anim. Ecol..

[bib0270] Stoffel M.A., Nakagawa S., Schielzeth H. (2017). rptR: repeatability estimation and variance decomposition by generalized linear mixed-effects models. Methods Ecol. Evol..

[bib0275] Stoye S., Porter M.A., Dawkins M.S. (2012). Synchronized lying in cattle in relation to time of day. Livest. Sci..

[bib0280] Sumner K.M., McCabe C.M., Nunn C.L. (2018). Network size, structure, and pathogen transmission: a simulation study comparing different community detection algorithms. Behaviour.

[bib0285] Turner J., Bowers R.G., Clancy D., Behnke M.C., Christley R.M. (2008). A network model of *E. coli* O157 transmission within a typical UK dairy herd: the effect of heterogeneity and clustering on the prevalence of infection. J. Theor. Biol..

[bib0290] VanderWaal K.L., Ezenwa V.O. (2016). Heterogeneity in pathogen transmission: mechanisms and methodology. Funct. Ecol..

[bib0295] VanderWaal K., Enns E.A., Picasso C., Packer C., Craft M.E. (2016). Evaluating empirical contact networks as potential transmission pathways for infectious diseases. J. R. Soc. Interface.

[bib0300] Wilson-Aggarwal J.K., Ozella L., Tizzoni M., Cattuto C., Swan G.J.F., Moundai T., Silk M.J., Zingeser J.A., McDonald R.A. (2019). High-resolution contact networks of free-ranging domestic dogs *Canis familiaris* and implications for transmission of infection. PLoS Negl. Trop. Dis..

[bib0305] Xie X., Li Y., Chwang A.T.Y., Ho P.L., Seto W.H. (2007). How far droplets can move in indoor environments - revisiting the Wells evaporation-falling curve. Indoor Air.

