# Supplementary material for: Spatial and temporal variation in proximity networks of commercial dairy cattle in Great Britain
Source: Prev Vet Med. 2021 Sep;194:105443. doi: 10.1016/j.prevetmed.2021.105443 (PMC8385416; doi:10.1016/j.prevetmed.2021.105443)
Supplement: Supplementary file 1 [file mmc1.docx]

**Spatial and temporal variation in proximity networks of commercial dairy cattle in Great Britain**

Helen R. Fielding, Matthew J. Silk, Trevelyan J. McKinley, Richard J. Delahay, Jared K. Wilson-Aggarwal, Laetitia Gauvin, Laura Ozella, Ciro Cattuto, and Robbie A. McDonald

**Supplementary materials**

**Figure S1.** Example of the cow-cow contacts (post-cleaning as defined in methods) recorded from proximity tags in one group of cows. Two tags (shown by green and purple lines) recorded disproportionate numbers of contacts over a short time period compared to other tags in the same deployment (all other coloured lines), such that this data is likely to be erroneous and not biologically plausible. All data recorded by proximity tags that recorded more contacts than 95% of the total contacts recorded by all tags within a 30-minute time frame were removed from the study.

**
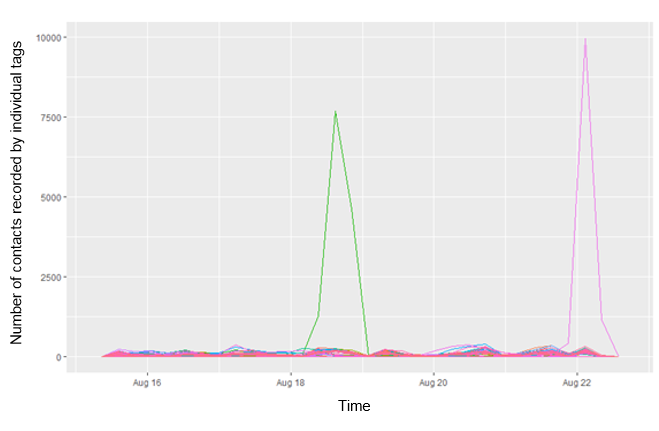
**

**Figure S2:** Variation in the proportion of two-hour periods a dyad spent in contact over each 24-hour period of the study period, compared to the mean proportion of two-hour periods they spent in contact. Each point represents a dyad and is coloured by the P value comparing it to a random distribution, with P < 0.025 as triangles and P > 0.025 as crosses. Dyads more consistent than random exhibit a range of mean contact times.

*
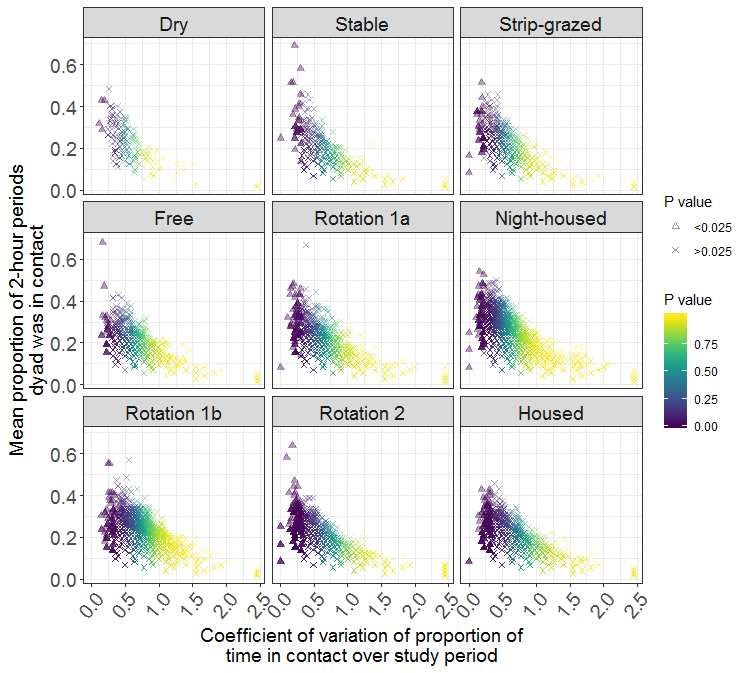
*

**Figure S3.** Community structure for unweighted, unfiltered Full networks and Spatial networks (Buildings, Pasture and Split) for all study groups (NF) and for those networks filtered by removing edge weights below the 50^th^ (F50), 75^th^, (F75) and 90^th^ percentile (F90) of unfiltered edge weights. a) shows the number of communities detected by the fast-greedy algorithm (calculated in ‘igraph’) and b) shows the proportion of the group in the largest detected community. Study groups are in order of ascending group size.

***
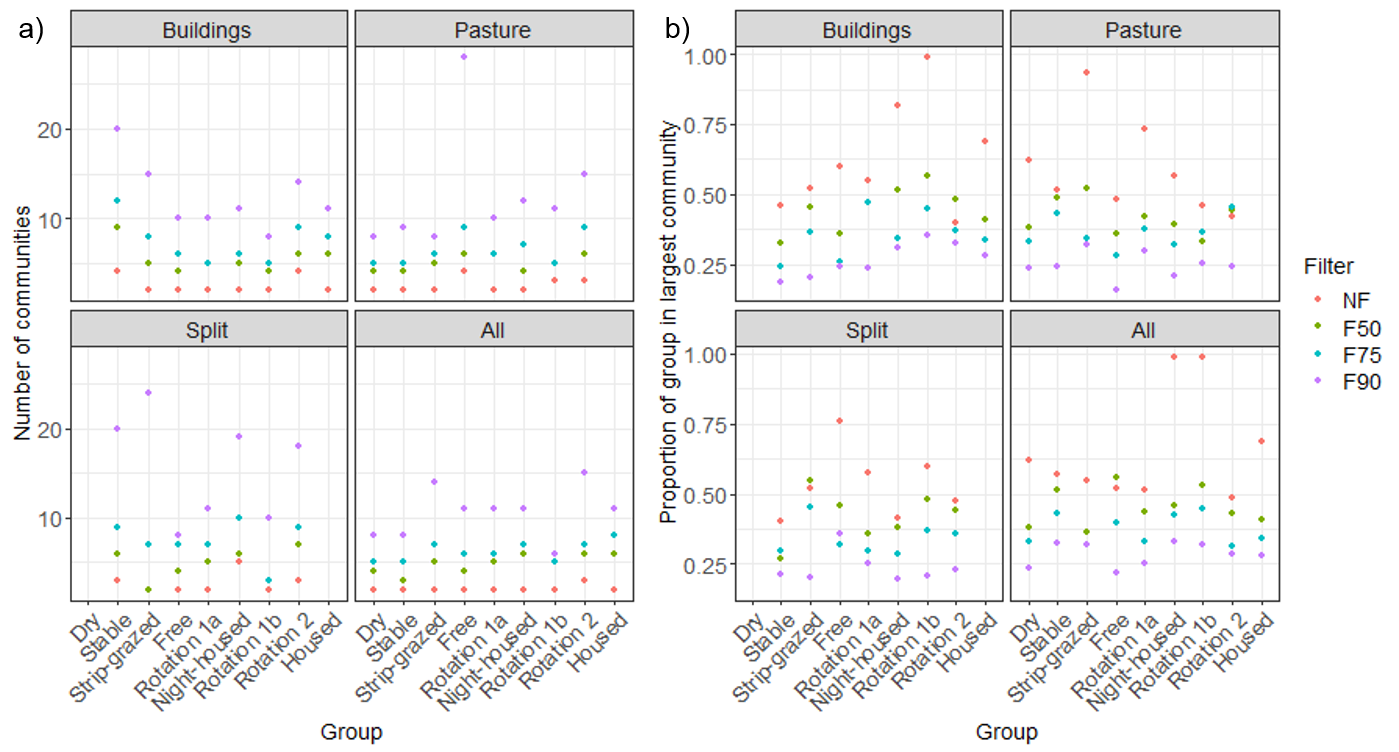
***

**Figure S4.** Consistency of community membership over time in contact networks of nine groups of dairy cattle split by time (day and night) and space (buildings, pasture, split). Categories (i.e. Split Night) were excluded if there were fewer than three comparable networks to define repeatability, therefore values for some networks are not present. Consistency of community membership was assessed by calculating the repeatability of cow-cow pairs being in the same community in sequential temporospatial networks. Repeatability values near 0, indicate a lack of stability in community membership, whereas values of 1 indicate perfectly stable community membership. Boxplots show the distribution of repeatability values from random networks and triangles and crosses represent observed repeatability values. Triangles indicate repeatability values that lie above 95% of random network values and crosses indicate repeatability values within 95% of random network values. Boxplots show the median, 25th and 75th percentiles of repeatability values calculated on randomised data (n = 4999) and the upper and lower whiskers extend to the largest or smallest value no further than 1.5 times the interquartile range, data beyond this range are not plotted. Groups are arranged by ascending group size.

**Figure S5.** Relative modularity values for each deployment for Full and Spatial networks (Buildings, Pasture and Split). The modularity was calculated for each of these additionally on three filtered networks removing edge weights less than the 50^th^ (F50), 75^th^, (F75) and 90^th^ percentile (F90) of unfiltered edge weights. Up and down facing triangles facing triangles respectively represent values higher and lower than 95% random values, crosses represent non-significance. Due to the resolution limit problem associated with modularity-based community detection, comparison between filtered networks with varying link densities should be avoided (Chen et al., 2018). Networks with fewer edges are likely to find smaller or more weakly connected communities therefore modularity should only be compared between networks filtered at the same level.

***
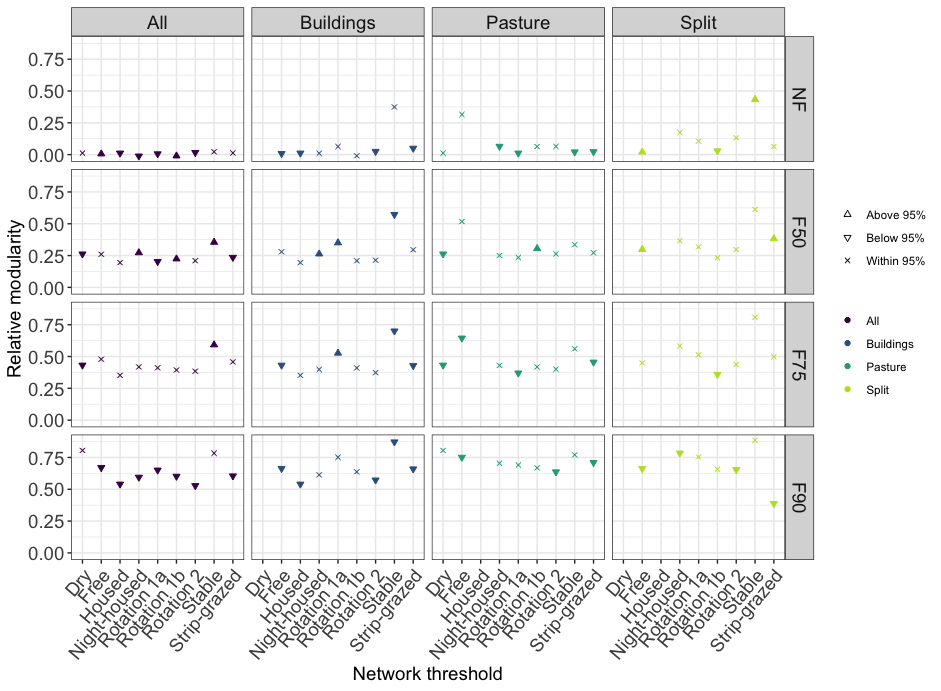
***

**Reference**

Chen, T., Singh, P., Bassler, K.E., 2018. Network community detection using modularity density measures. J. Stat. Mech. Theory Exp. https://doi.org/doi.org/10.1088/1742-5468/aabfc8

**Table S1.** Details of farm management and data collected from nine groups of cattle on seven dairy farms in Cornwall in Summer and Autumn 2018. Details include breed of cattle, grazing management, milking routine, group sizes, number of collars with GPS and proximity sensors that were deployed, and data quality

| Deployment name | Mean contact rate per dyad per hour | Mean minutes cow-cow contact per day | Edge density of Full network |
| --- | --- | --- | --- |
|  |  |  |  |
| Night-housed | 0.27 | 33.23 | 0.97 |
| Strip-grazed | 0.20 | 23.78 | 0.95 |
| Free | 0.32 | 42.63 | 0.98 |
| Rotation 1a | 0.18 | 21.50 | 0.95 |
| Rotation 1b | 0.27 | 39.46 | 0.99 |
| Dry | 0.29 | 36.02 | 0.93 |
| Housed | 0.14 | 18.69 | 0.92 |
| Rotation 2 | 0.15 | 17.69 | 0.92 |
| Stable | 0.13 | 12.07 | 0.93 |
| Mean | 0.22 | 27.23 | 0.95 |

**Table S2.** Correlation values for comparing Spatial networks from quadratic assignment procedure analysis. Analysis was performed on unfiltered networks and networks filtered by removing edge weights less than the 50^th^ (F50), 75^th^, (F75) and 90^th^ percentile (F90) of unfiltered edge weights. Grey shading indicates correlation scores significantly different from random (P < 0.05).

| Farm | Metric | Networks compared | | | | | | | | | | | |
| --- | --- | --- | --- | --- | --- | --- | --- | --- | --- | --- | --- | --- | --- |
|  |  | Pasture and Buildings | | | | Pasture and Split | | | | Buildings and Split | | | |
|  | Threshold | NF | F50 | F75 | F90 | NF | F50 | F75 | F90 | NF | F50 | F75 | F90 |
| Night-housed | *P* value | 0.808 | 0.984 | 0.324 | 0.154 | 0.519 | 0.678 | 0.561 | 0.924 | 0.767 | 0.826 | 0.791 | 0.780 |
|  | R squared | 0.0000 | 0.0000 | 0.0004 | 0.0007 | 0.0001 | 0.0001 | 0.0001 | 0.0000 | 0.0001 | 0.0000 | 0.0000 | 0.0000 |
| Strip-grazed | *P* value | <0.001 | <0.001 | <0.001 | <0.001 | <0.001 | <0.001 | <0.001 | <0.001 | <0.001 | <0.001 | <0.001 | <0.001 |
|  | R squared | 0.0972 | 0.0860 | 0.0574 | 0.0475 | 0.0942 | 0.0940 | 0.0649 | 0.0457 | 0.3096 | 0.2961 | 0.2289 | 0.1754 |
| Free | *P* value | 0.344 | 0.323 | 0.377 | 0.212 | 0.017 | 0.006 | 0.015 | 0.045 | <0.01 | <0.01 | <0.01 | 0.005 |
|  | R squared | 0.0013 | 0.0013 | 0.0009 | 0.0015 | 0.0092 | 0.0100 | 0.0081 | 0.0044 | 0.0580 | 0.0465 | 0.0268 | 0.0102 |
| Rotation 1a | *P* value | <0.001 | <0.001 | 0.002 | 0.041 | <0.001 | <0.001 | <0.001 | <0.001 | <0.001 | <0.001 | <0.001 | <0.001 |
|  | R squared | 0.0135 | 0.0154 | 0.0069 | 0.0020 | 0.0607 | 0.0549 | 0.0423 | 0.0169 | 0.0395 | 0.0374 | 0.0295 | 0.0355 |
| Rotation 1b | *P* value | <0.001 | <0.001 | <0.001 | <0.001 | <0.001 | <0.001 | <0.001 | <0.001 | <0.001 | <0.001 | <0.001 | <0.001 |
|  | R squared | 0.0640 | 0.0587 | 0.0397 | 0.0225 | 0.0589 | 0.0540 | 0.0430 | 0.0315 | 0.1012 | 0.0912 | 0.0746 | 0.0510 |
| Rotation 2 | *P* value | <0.001 | <0.001 | <0.001 | <0.001 | <0.001 | <0.001 | <0.001 | 0.019 | <0.001 | <0.001 | <0.001 | <0.001 |
|  | R squared | 0.0113 | 0.0110 | 0.0073 | 0.0028 | 0.0041 | 0.0040 | 0.0029 | 0.0018 | 0.0997 | 0.0935 | 0.0773 | 0.0560 |
| Stable | *P* value | 0.961 | 0.944 | 0.463 | 0.980 | 0.585 | 0.548 | 0.501 | 0.604 | 0.873 | 0.795 | 0.869 | 0.844 |
|  | R squared | 0.0000 | 0.0000 | 0.0009 | 0.0000 | 0.0005 | 0.0006 | 0.0006 | 0.0003 | 0.0000 | 0.0001 | 0.0001 | 0.0001 |

**Table S3.** Relative modularity values for communities detected by the fast-greedy algorithm on binary (unweighted) Full networks and Spatial networks. To analyse stronger ties, we filtered the networks by removing edge weights below the 50^th^ (F50), 75^th^ (F75), and 90^th^ (F90) percentiles. NF indicates the unfiltered network. LCI = Lower bound of random networks (2.5%) UCI = Upper bound of random networks (97.5%) Qrel = relative modularity. Dark grey shading of the cell indicates the observed relative modularity was lower than 2.5% lowest values of relative modularity calculated on 4999 randomised networks, light grey shading indicates observed values were higher than 97.5% of values on random networks.

|  | Filter | NF | F50 | F75 | F90 | NF | F50 | F75 | F90 | NF | F50 | F75 | F90 | NF | F50 | F75 | F90 |
| --- | --- | --- | --- | --- | --- | --- | --- | --- | --- | --- | --- | --- | --- | --- | --- | --- | --- |
|  | Location | Pasture | | | | Buildings | | | | Split | | | | All | | | |
| Night-housed | LCI | 0.0699 | 0.2487 | 0.4281 | 0.6751 | 0.0096 | 0.1772 | 0.3447 | 0.6128 | 0.1551 | 0.3356 | 0.5567 | 0.7896 | 0.0071 | 0.1767 | 0.3455 | 0.6106 |
|  | UCI | 0.0964 | 0.3119 | 0.5156 | 0.7492 | 0.0199 | 0.2307 | 0.4231 | 0.6948 | 0.2063 | 0.4147 | 0.6436 | 0.8455 | 0.0177 | 0.2306 | 0.4236 | 0.6920 |
|  | Qrel | 0.0651 | 0.2505 | 0.4296 | 0.7041 | 0.0103 | 0.2627 | 0.3984 | 0.6134 | 0.1738 | 0.3668 | 0.5821 | 0.7846 | -0.0111 | 0.2722 | 0.4183 | 0.5948 |
| Strip-grazed | LCI | 0.0355 | 0.2517 | 0.4751 | 0.7294 | 0.0539 | 0.2676 | 0.4905 | 0.7331 | 0.0506 | 0.2768 | 0.4784 | 0.7336 | 0.0097 | 0.2379 | 0.4492 | 0.7150 |
|  | UCI | 0.0599 | 0.3340 | 0.5782 | 0.8132 | 0.0825 | 0.3475 | 0.5939 | 0.8156 | 0.0786 | 0.3615 | 0.5820 | 0.8165 | 0.0335 | 0.3164 | 0.5542 | 0.7993 |
|  | Qrel | 0.0231 | 0.2718 | 0.4561 | 0.7103 | 0.0501 | 0.2954 | 0.4285 | 0.6601 | 0.0636 | 0.3824 | 0.4978 | 0.3880 | 0.0130 | 0.2359 | 0.4579 | 0.6048 |
| Free | LCI | 0.2627 | 0.4816 | 0.6746 | 0.8677 | 0.0187 | 0.2278 | 0.4438 | 0.6988 | -0.0203 | 0.2204 | 0.4259 | 0.6934 | -0.0205 | 0.2176 | 0.4249 | 0.6898 |
|  | UCI | 0.3438 | 0.5801 | 0.7610 | 0.9423 | 0.0379 | 0.3007 | 0.5440 | 0.7816 | 0.0199 | 0.2927 | 0.5256 | 0.7763 | 0.0055 | 0.2888 | 0.5259 | 0.7745 |
|  | Qrel | 0.3155 | 0.5174 | 0.6451 | 0.7515 | 0.0085 | 0.2794 | 0.4320 | 0.6635 | 0.0204 | 0.2985 | 0.4497 | 0.6631 | 0.0056 | 0.2599 | 0.4787 | 0.6705 |
| Rotation 1a | LCI | 0.0261 | 0.2152 | 0.4039 | 0.6710 | 0.0498 | 0.2339 | 0.4295 | 0.6901 | 0.0961 | 0.3055 | 0.4876 | 0.7511 | 0.0172 | 0.2104 | 0.3929 | 0.6653 |
|  | UCI | 0.0404 | 0.2795 | 0.4987 | 0.7511 | 0.0699 | 0.2994 | 0.5249 | 0.7663 | 0.1389 | 0.3846 | 0.5817 | 0.8179 | 0.0316 | 0.2756 | 0.4877 | 0.7466 |
|  | Qrel | 0.0122 | 0.2348 | 0.3705 | 0.6908 | 0.0635 | 0.3502 | 0.5264 | 0.7516 | 0.1055 | 0.3179 | 0.5134 | 0.7537 | 0.0063 | 0.2035 | 0.4115 | 0.6509 |
| Rotation 1b | LCI | 0.0609 | 0.2330 | 0.3950 | 0.6609 | -0.0115 | 0.1721 | 0.3392 | 0.6027 | 0.0363 | 0.2004 | 0.3644 | 0.6319 | -0.0116 | 0.1699 | 0.3375 | 0.6051 |
|  | UCI | 0.0803 | 0.2921 | 0.4803 | 0.7380 | 0.0024 | 0.2240 | 0.4168 | 0.6870 | 0.0489 | 0.2555 | 0.4462 | 0.7107 | -0.0113 | 0.2233 | 0.4158 | 0.6898 |
|  | Qrel | 0.0635 | 0.3050 | 0.4180 | 0.6683 | -0.0094 | 0.2089 | 0.4103 | 0.6365 | 0.0297 | 0.2322 | 0.3598 | 0.6569 | -0.0102 | 0.2238 | 0.3938 | 0.6021 |
| Rotation 2 | LCI | 0.0628 | 0.2168 | 0.3855 | 0.6544 | 0.0401 | 0.1956 | 0.3528 | 0.6197 | 0.0973 | 0.2727 | 0.4339 | 0.7028 | 0.0299 | 0.1824 | 0.3462 | 0.6037 |
|  | UCI | 0.0837 | 0.2710 | 0.4701 | 0.7294 | 0.0524 | 0.2477 | 0.4328 | 0.7012 | 0.1360 | 0.3379 | 0.5191 | 0.7729 | 0.0400 | 0.2337 | 0.4236 | 0.6867 |
|  | Qrel | 0.0648 | 0.2630 | 0.4005 | 0.6369 | 0.0250 | 0.2137 | 0.3732 | 0.5727 | 0.1321 | 0.2973 | 0.4373 | 0.6542 | 0.0167 | 0.2096 | 0.3843 | 0.5286 |
| Stable | LCI | 0.0259 | 0.2706 | 0.4829 | 0.7263 | 0.3459 | 0.6126 | 0.7558 | 0.8786 | 0.2982 | 0.5478 | 0.7405 | 0.8771 | 0.0166 | 0.2639 | 0.4765 | 0.7296 |
|  | UCI | 0.0517 | 0.3597 | 0.5885 | 0.8245 | 0.4477 | 0.7145 | 0.8513 | 1.0000 | 0.3915 | 0.6558 | 0.8347 | 1.0000 | 0.0441 | 0.3508 | 0.5841 | 0.8238 |
|  | Qrel | 0.0213 | 0.3350 | 0.5605 | 0.7702 | 0.3742 | 0.5731 | 0.7007 | 0.8721 | 0.4334 | 0.6128 | 0.8084 | 0.8853 | 0.0223 | 0.3545 | 0.5919 | 0.7837 |
| Dry | LCI | -0.0002 | 0.2831 | 0.5121 | 0.7132 | – | – | – | – | – | – | – | – | -0.0023 | 0.2844 | 0.5134 | 0.7159 |
|  | UCI | 0.0303 | 0.4056 | 0.6639 | 0.9158 | – | – | – | – | – | – | – | – | 0.0303 | 0.4060 | 0.6643 | 0.9192 |
|  | Qrel | 0.0119 | 0.2624 | 0.4324 | 0.8054 | – | – | – | – | – | – | – | – | 0.0119 | 0.2624 | 0.4324 | 0.8054 |
| Housed | LCI | – | – | – | – | 0.0314 | 0.1789 | 0.3374 | 0.6004 | – | – | – | – | 0.0315 | 0.1794 | 0.3368 | 0.6001 |
|  | UCI | – | – | – | – | 0.0413 | 0.2280 | 0.4134 | 0.6823 | – | – | – | – | 0.0416 | 0.2285 | 0.4121 | 0.6829 |
|  | Qrel | – | – | – | – | 0.0120 | 0.1945 | 0.3521 | 0.5407 | – | – | – | – | 0.0120 | 0.1945 | 0.3521 | 0.5407 |
